# Microfluidic Optimization of PEI-Lipid Hybrid Nanoparticles for Efficient DNA Delivery and Transgene Expression

**DOI:** 10.3390/pharmaceutics17040454

**Published:** 2025-04-01

**Authors:** Mahboubeh Hosseini-Kharat, Anthony Wignall, Zelalem A. Mekonnen, Ben S.-Y. Ung, Bradley Chereda, Kristen E. Bremmell, Branka Grubor-Bauk, Clive A. Prestidge

**Affiliations:** 1 Clinical and Health Sciences, Centre for Pharmaceutical Innovation, University of South Australia, Adelaide, SA 5000, Australia; mitra.hosseini_kharat@mymail.unisa.edu.au (M.H.-K.); anthony.wignall@unisa.edu.au (A.W.); kristen.bremmell@unisa.edu.au (K.E.B.); 2 Viral Immunology Group, Basil Hetzel Institute for Translational Health Research, Adelaide Medical School, University of Adelaide, Woodville South, SA 5011, Australia; zelalem.mekonnen@adelaide.edu.au (Z.A.M.); branka.grubor@adelaide.edu.au (B.G.-B.); 3 Quality Use of Medicines and Pharmacy Research Centre, University of South Australia City East Campus, Frome Rd., Adelaide, SA 5000, Australia; ben.ung@unisa.edu.au; 4 Centre for Cancer Biology, University of South Australia and SA Pathology, Adelaide, SA 5000, Australia; bradley.chereda@unisa.edu.au

**Keywords:** lipid nanoparticles (LNPs), polyethyleneimine (PEI), DNA delivery, microfluidic mixing, transgene expression, luciferase assays, GFP expression, lipid-polymer hybrid systems, gene therapy

## Abstract

**Background**: Lipid nanoparticles (LNPs) and polyethyleneimine (PEI) have independently been used for DNA complexation and delivery. However, non-ideal gene delivery efficiency and toxicity have hindered their clinical translation. We developed DNA-PEI-LNPs as a strategy to overcome these limitations and enhance DNA delivery and transgene expression. **Methods**: Three microfluidic mixing protocols were evaluated: (i) LNPs without PEI, (ii) a single-step process incorporating PEI in the organic phase, and (iii) a two-step process with DNA pre-complexed with PEI before LNP incorporation. The influence of DNA/PEI ratios (1:1, 1:2, 1:3) and DNA/lipid ratios (1:10, 1:40) on particle properties and delivery efficiency was examined. **Results**: In luciferase formulations, higher DNA/lipid ratios (1:40) produced smaller particles (136 nm vs. 188 nm) with improved cellular uptake (77% vs. 50%). The two-step method with higher DNA/PEI ratios improved transfection efficiency, with LNP-Luc/PEI 1:3 (40) achieving ~1.9 × 10^6^ relative light units (RLU) in luciferase expression. In green fluorescent protein (GFP) studies, LNP-GFP/PEI 1:3 (40) showed ~23.8% GFP-positive cells, nearly twofold higher than LNP-GFP (40) at ~12.6%. **Conclusions**: These results demonstrate the capability of microfluidic-prepared DNA-PEI-LNPs to improve DNA delivery and transgene expression through optimized formulation strategies and selection of appropriate preparation methods.

## 1. Introduction

Gene therapy represents a therapeutic strategy designed to combat genetic diseases by introducing nucleic acids into target cells to add, modify, or regulate gene expression [1,2]. Critical to this therapeutic strategy are delivery systems that can transport nucleic acids into cells safely while maintaining both genetic material stability and cell function. Non-viral vectors (e.g., LNPs and cationic polymers like PEI) have become increasingly important as they overcome common limitations of viral vectors, such as immune responses and limited cargo capacity [3,4,5,6]. LNPs especially have drawn attention as nucleic acid carriers, offering both biocompatibility and protection against enzymatic destruction [7,8,9] Their protective capabilities stem from two main features: efficient nucleic acid encapsulation within their lipid matrix and optimized surface charge characteristics [10,11].

However, low transfection efficiency and poor endosomal escape have driven the development of strategies to improve delivery. In this regard, PEI—a cationic polymer that provides high transfection efficiency—has been investigated for increasing the gene-delivery potential of LNPs [12]. The reason PEI is so efficient at carrying genes is that it has a high cationic charge density, which allows nucleic acids to condense into small molecules that are readily taken up by the cell [13]. In addition, PEI’s ‘proton sponge’ effect helps genes escape from endosomes and enter the cytoplasm [14,15], though some recent studies question parts of this mechanism [16].

Bus et al. [17] described three main ways cationic polyplexes escape from endosomes. First, the “proton sponge effect” occurs when polymers act as a buffer inside the endosome, increasing pressure until the membrane bursts. Second, polyplexes can disrupt the endosomal membrane directly, forming small holes. Third, free polymer chains (not part of the complex) can interact with the membrane, making it more permeable. The main disadvantage of PEI comes from its cellular toxicity, which varies with its molecular weight and branching structure. To reduce this toxicity, researchers have created strategies (e.g., modifying PEI or combining it with lipids to form hybrid delivery systems) [18,19].

A range of PEI-lipid hybrid systems, with molecular weights between 600 Da and 25 kDa, have been developed using preparation methods such as ethanol dilution [20], lipid hydration [21], microfluidic focusing [22], and phase inversion [12]. Plasmid DNA [12,19,23,24] and siRNA [21,22,25] have been delivered using these methods. High-molecular-weight PEI (25 kDa) exhibits strong transfection efficiency but raises toxicity concerns [19,20]. Lower molecular weight PEI with molecular sizes between 600 Da and 800 Da demonstrates reduced cytotoxic effects yet exhibits decreased stability in complexes [22,24]. Recent studies demonstrated that intermediate-weight PEI (1.8–2 kDa) formulations found a balance between transfection efficiency and biocompatibility [12,25].

PEI-functionalized lipid nanocapsules for co-delivering genes and drugs showed a 2.8-fold higher transfection efficiency than free PEI/pDNA, with stable complexes and low cytotoxicity, enabling drug co-delivery (e.g., paclitaxel) [12]. Lipopolyplexes combining cationic liposomes and PEI improved gene transfection efficiency and remained stable post-nebulization which qualify them for aerosol delivery [21]. PEI-based lipopolyplexes were also tested in cancer models for anticancer gene therapy, offering systemic stability, reduced cytotoxicity, and efficient intravenous delivery of siRNA, miRNA, antisense oligonucleotides, or tumour-suppressor genes [26]. Our group previously studied PEI (25 kDa) and polyamidoamine (PAMAM)-based lipopolyplexes for gene delivery applications. PEI and PAMAM attached to lipid bilayers but showed distinct membrane disruption behaviours in which PEI generated nanoscale disruptions and PAMAM created circular holes. The PAMAM-based lipopolyplexes demonstrated better cellular uptake but presented greater toxicity compared to PEI-based lipopolyplexes [27].

Microfluidics has emerged as an effective method to deal with the preparation challenges of PEI-lipid hybrid systems and achieve better control over formulation properties [28]. This approach permits precise regulation of the preparation process, maintaining uniformity in particle size, composition, and reproducibility [29,30,31]. Microfluidic mixing helps for overcoming the limitations of conventional batch methods by allowing better coordination of how lipids and polymers combine to form stable nanoparticles with high encapsulation efficiency. Maeki et al. [32] previously used microfluidics with high molecular weight PEI (10 kDa) and large plasmid DNA (15 kbp) at a 1:1 pDNA-PEI ratio. In contrast, our study centers on lower molecular weight PEI (1.8 kDa) and tests different ways of incorporating PEI, including pre-complexed formation and single-phase mixing. We optimize DNA/PEI ratios (1:1, 1:2, 1:3) to balance transfection efficiency, cytotoxicity, and stability.

## 2. Materials and Methods

### 2.1. Plasmid DNA Purification

Plasmid DNA encoding luciferase and GFP was transformed into *Escherichia coli* DH5α competent cells using a heat shock method and cultured on Lysogeny Broth (LB) agar plates supplemented with the appropriate antibiotics. Single colonies were picked and grown in LB media with antibiotics at 37 °C with shaking overnight. Plasmid DNA was purified using the HiSpeed Plasmid Midi Kit (QIAGEN, Hilden, Germany) [33], according to the manufacturer’s instructions. This method includes an optimized alkaline lysis procedure followed by binding of plasmid DNA to the HiSpeed Tips under low-salt and low-pH conditions. The DNA was subsequently eluted in a high-salt buffer and precipitated with isopropanol. The final DNA yield and purity were measured using a NanoDrop Lite Spectrophotometer (Thermo Fisher Scientific, Wilmington, DE, USA).

### 2.2. Formulation Preparation

**Figure 1:**
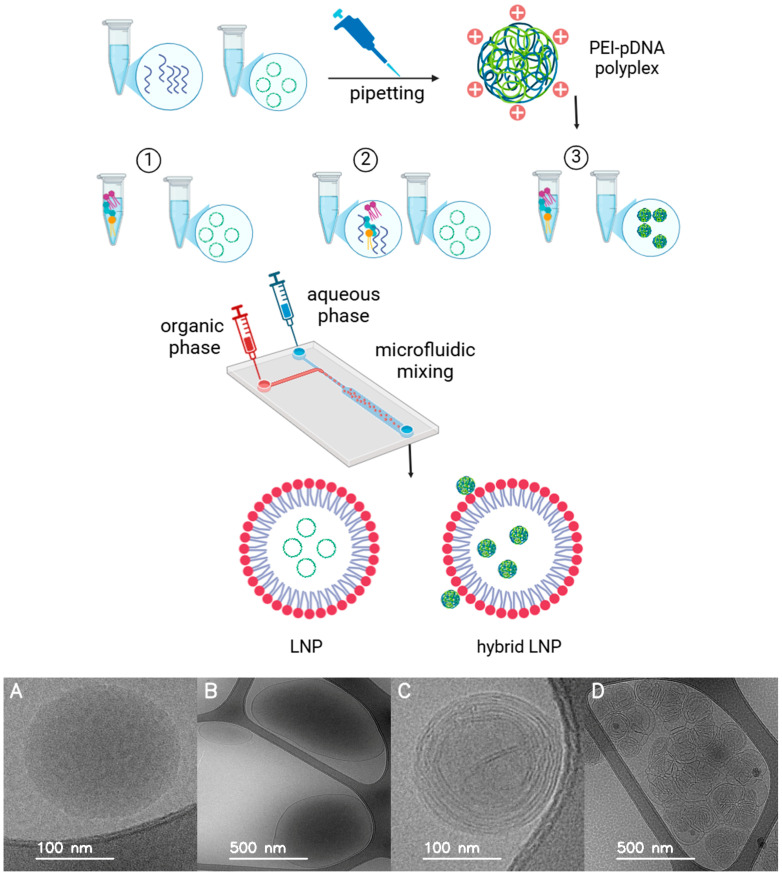
**Top**: Schematic representation of LNP and hybrid LNP-PEI preparation using microfluidic mixing. (**1**) LNPs: DNA and lipids are mixed directly. (**2**) One-step hybrid LNP-PEI: PEI is added to the organic phase. (**3**) Two-step hybrid LNP-PEI: DNA is pre-complexed with PEI before mixing with lipids. Colour scheme: green circular, coiled structures indicate pDNA, blue strands represent PEI, and red circles represent lipid head groups (outer surface). Created with BioRender.com. **Bottom** (Supplementary Materials Figure S1): Cryo-TEM images of representative LNP formulations showing structural variations: (**A**) LNP-Luc (40), (**B**,**C**) LNP-Luc/PEI (40, Org), with (**C**) displaying onion-like multilamellar structure, and (**D**) LNP-Luc/PEI 1:3 (40), also showing multilamellar structures.

Plasmid DNA formulations were prepared using LNPs and PEI under varying DNA/Lipid and DNA/PEI ratios. The lipid mixture—1,2-dioleoyl-3-trimethylammonium-propane (DOTAP) (50%), 1,2-distearoyl-sn-glycero-3-phosphocholine (DSPC) (10%), cholesterol (38.5%), and 1,2-dimyristoyl-rac-glycero-3-methoxypolyethylene glycol-2000 (DMG-PEG 2000) (1.5%) (Avanti Research™, A Croda Brand, Alabaster, AL, USA)—was dissolved in ethanol to form the organic phase. PEI (branched, average Mw 1800, 50 wt. % in H_2_O, Sigma-Aldrich, St. Louis, MO, USA) was used for hybrid formulations. All formulations were prepared at a final volume of 1 mL, consisting of 250 µL of organic phase and 750 µL of aqueous phase (1× PBS, pH 7.4, 137 mM NaCl, 2.7 mM KCl, 10 mM phosphate, prepared by diluting 10× PBS (Sigma-Aldrich) with nuclease-free water) at a flow rate ratio of 3:1 and a total flow rate of 12 mL/min using the NanoAssemblr™ Ignite™ microfluidic mixing system (Cytiva, Vancouver, BC, Canada). For formulation, PEI stock solution (1.0 µg/µL in nuclease-free water) was either diluted in 1× PBS (pH 7.4, 137 mM NaCl, 2.7 mM KCl, 10 mM phosphate) to match the DNA volume in the aqueous phase (for pre-complexed formulations) or added directly to the organic phase (for single-phase formulations). We selected the 3:1 aqueous-to-organic volumetric flow rate ratio, known to optimize nanoparticle size and encapsulation efficiency, as described in several studies [31,34]. Also, the total flow rate was adjusted to 12 mL/min to achieve uniform mixing and reproducibility [35]. The three main formulation types were prepared as follows (Figure 1, top):

1. LNP formulations without PEI: For LNP-Luc (10) and LNP-Luc (40), we mixed plasmid DNA with the aqueous phase and dissolved the lipid mixture in ethanol to form the organic phase. The phases were combined via one-step microfluidic mixing (NanoAssemblr), producing LNPs encapsulating DNA.
2. LNP formulations with PEI in the organic phase (one-step method): For LNP-Luc/PEI (10, Org) and LNP-Luc/PEI (40, Org), we added PEI to the organic phase with lipids and mixed it with the DNA-containing aqueous phase using one-step microfluidic mixing.
3. LNP formulations with pre-complexed PEI-DNA in the aqueous phase (two-step method): For LNP-Luc/PEI 1:1, 1:2, and 1:3 (10) and (40), we pre-complexed PEI with DNA in the aqueous phase at ratios of 1:1, 1:2, or 1:3 by dropwise addition and 30-min incubation. The complexed solution was mixed with the organic lipid phase via two-step microfluidic mixing.

### 2.3. Characterization of Formulations

We measured LNP hydrodynamic diameter (HD), polydispersity index (PDI), and zeta potential using a Zetasizer Nano ZS machine (Malvern, Worcestershire, UK). Encapsulation efficiency (*EE*) for formulations without PEI was determined using the Quant-iT™ dsDNA Assay Kit, High Sensitivity (HS) (Invitrogen™, Waltham, MA, USA). LNP samples were diluted in Tris-EDTA (TE) Buffer (Invitrogen™) to achieve DNA concentrations in the range of 0.2–100 ng. Samples were divided into two aliquots: one treated with Triton™ X-100 (0.5% *v*/*v*, Sigma-Aldrich) to release encapsulated DNA, and the other left untreated to measure free DNA. The Quant-iT™ reagent was added, incubated for 5 min, and fluorescence was measured using a microplate reader at 485 nm excitation and 500–550 nm emission. Using Equation (1), we determined that the encapsulation efficiency was 85.0 ± 0.5% for the lower lipid concentration formulation (LNP-Luc (10)) and 90.0 ± 0.5% for the higher lipid concentration formulation (LNP-Luc (40)).

(1) $$EE\;(\%)=\left(\;\frac{Lysed\;LNP\;fluorescence-Free\;DNA\;fluorescence}{Lysed\;LNP\;fluorescence}\right)\times 100$$

For formulations containing PEI, because PEI interferes with fluorescence signals in the Quant-iT™ dsDNA HS Assay, an alternative method was used. Encapsulation efficiency was assessed via differential centrifugation. A 100 µL volume of each sample was split into two tubes: Tube 1 (total DNA, lysed with 0.5% Triton X-100) and Tube 2 (centrifuged at 20,000× *g*, 4 °C, 30–60 min) to separate free DNA. The supernatant (Tube 3) contained free DNA, while the pellet was lysed with 0.5% Triton X-100 to release encapsulated DNA. DNA concentrations were measured via Nanodrop, and *EE*% was calculated as:

(2) $$EE\;(\%)=\left(\frac{Total\;DNA-Free\;DNA\;}{Total\;DNA\;}\right)\times 100$$

*EE* for formulations prepared using the one-step method was 90.2 ± 1.1% (range: 89–92%), while the two-step method achieved higher encapsulation, with EE values of 98.1 ± 0.8% (range: 97–99%).

### 2.4. Cellular Uptake Study

Formulations were labelled with Atto 532 Dioleoylphosphatidylethanolamine (DOPE) (0.2 mol% of total lipid) to visualize cellular uptake. HEK 293 cells were seeded into 96-well plates at a density of 4 × 10^4^ cells per well 24 h prior to the experiment. We used HEK 293 cells because of their well-documented transfection efficiency, simple maintenance, and human origin, positioning them as a widely used model for nanoparticle-mediated gene delivery studies [36]. Their use in lipid nanoparticle-mediated mRNA delivery (e.g., for vaccine development) has been widely documented [37], and they commonly serve in transfection protocols for gene expression analysis [38]. Cells were incubated with formulations containing 0.2 µg DNA per well for 4 h at 37 °C. After incubation, cells were washed with phosphate-buffered saline (PBS, Sigma Aldrich), detached using trypsin-EDTA solution (Sigma Aldrich), and then resuspended in PBS for flow cytometry analysis. Flow cytometry measurements were acquired using an LSRFortessa™ (BD Biosciences, San Jose, CA, USA). Light-scatter and Atto-532 were detected using the 488 nm (50 mW) and 561 nm (50 mW) lasers, respectively. Data were analysed using FACS Diva 8.0.3. For confocal microscopy imaging, cells were seeded into Ibidi 8-well plates at the same density. After 4 h of incubation with formulations containing 0.5 µg DNA per well, cells were stained to visualize key structures. The cell membrane was stained with Wheat Germ Agglutinin (WGA) Alexa Fluor 633 conjugate (Invitrogen™), nuclei were stained with 4′,6-diamidino-2-phenylindole (DAPI) in Fluoroshield™ mounting medium (Sigma Aldrich), and cells were fixed using 4% paraformaldehyde (PFA, Thermo Scientific Chemicals, Waltham, MA, USA). Confocal images were acquired using the LMS 710 Carl Zeiss confocal microscope (Oberkochen, Germany).

### 2.5. Luciferase Expression Assay

HEK 293 cells were seeded into a 96-well plate at a density of 3 × 10^4^ cells per well 24 h prior to the experiment. Cells were incubated with formulations containing 0.2 µg DNA per well for 48 h at 37 °C. After the incubation period, the medium was removed, and the cells were washed with PBS. Cell lysis was performed by adding 25 µL of lysis buffer from the Luciferase Assay System (Promega, Madison, WI, USA) to each well and incubating for 30 min at room temperature. The lysate was transferred to a white, opaque OptiPlate 96-well microplate (Perkin Elmer, Shelton, CT, USA). Then, 25 µL of luciferin reagent (Promega) was added to each well, and luminescence was measured using a plate reader.

### 2.6. Cell Viability Assay

HEK 293 cells were seeded in a 96-well plate at a density of 3 × 10^4^ cells per well and incubated for 24 h at 37 °C. Formulations containing 0.2 µg DNA per well were added, and cells were incubated for 4 h. After incubation, the medium was removed, and MTT [3-(4,5-Dimethylthiazol-2-yl)-2,5-Diphenyltetrazolium Bromide] (Invitrogen™) reagent (0.5 mg/mL) was added to each well, followed by incubation for 4 h at 37 °C. The formazan crystals formed were dissolved in dimethyl sulfoxide (DMSO, Sigma Aldrich) (100 µL per well), and absorbance was measured at 570 nm using a plate reader [39].

Calculation of % cell viability:

(3) $$Cell\;Viability\;\left(\%\right)=\frac{Mean\;Absorbance\;of\;Treated\;Cells}{Mean\;Absorbance\;of\;Control\;Cells}\times 100$$

### 2.7. GFP Expression Assay

HEK 293 cells were seeded into a 96-well blacsk/clear bottom microplate (Thermo Scientific™, Waltham, MA, USA) at a density of 3 × 10^4^ cells per well and incubated for 24 h at 37 °C. Formulations were added, and cells were incubated with the formulations for an additional 24 h. After incubation, a flow cytometer (LSRFortessa™, BD Biosciences, San Jose, CA, USA) was used to determine the proportion of cells expressing GFP and the ZEISS Celldiscoverer 7 system (ZEISS, Oberkochen, Germany) was used to assess fluorescence intensity.

### 2.8. Cryo-TEM Sample Preparation and Imaging

Cryogenic transmission electron microscopy (Cryo-TEM) was performed to visualize the morphology of LNP formulations. A 5 μL aliquot of the sample was applied to a glow-discharged 300-mesh copper grid for 30 s. The sample was vitrified using a mixture of liquid ethane and propane and maintained at −180 °C during imaging. Imaging was conducted using a Glacios 200 kV Cryo-TEM (Thermo Fisher Scientific, Waltham, MA, USA) equipped with a NANOSPRT15 camera (Thermo Fisher Scientific), under bright-field conditions at 120 kV. All reagents used were of analytical grade to avoid imaging artifacts.

### 2.9. Statistical Analysis

All data are presented as mean ± standard error of the mean (SEM) from at least three independent experiments. For comparisons between two groups, statistical significance was determined using unpaired two-tailed Student’s *t*-tests. For comparisons between three or more groups, one-way analysis of variance (ANOVA) was performed. Statistical significance was set at *p* < 0.05 and is indicated as follows: ns (*p* > 0.05), * (*p* < 0.05), ** (*p* < 0.01), *** (*p* < 0.001), and **** (*p* < 0.0001). All statistical analyses and graph generation were performed using GraphPad Prism version 10.4.1 (GraphPad Software, San Diego, CA, USA).

## 3. Results and Discussion

### 3.1. Design of Lipid Nanoparticles for Luciferase DNA Delivery

The formulations outlined in Table 1 were designed to evaluate the influence of DNA/Lipid and DNA/PEI ratios, as well as preparation methods, on the delivery of pDNA encoding luciferase. All formulations share a common lipid composition (DOTAP, DSPC, cholesterol, and (DMG-PEG 2000) optimized for stability and cellular uptake. The one-step NanoAssemblr direct method was used to prepare LNP-Luc (10) and LNP-Luc (40), where higher DNA/Lipid ratios (1:40) were hypothesized to enhance DNA encapsulation and delivery efficiency. We also investigated the role of DNA/PEI ratios (e.g., 1:1, 1:2, and 1:3) in PEI-containing formulations using both one-step and two-step preparation methods. The one-step method, where PEI is added directly to the organic phase, is simpler but may yield different physicochemical properties compared to the two-step method, which uses pre-complexing PEI with DNA. These design variations allow a systematic exploration of the effects of lipid composition, PEI content, and preparation method on the formulation’s potential for gene delivery.

### 3.2. Physico-Chemical Characterization of Luciferase DNA-Loaded LNPs

The analysis illustrated in Table 1 shows how particle size, PDI, and zeta potential vary with the DNA/Lipid ratio, the addition of PEI, and the preparation method. Higher DNA/Lipid ratios (e.g., 1:40) generally produced smaller particles than lower ratios (e.g., 1:10). As shown in previous studies with lipid nanoparticle systems, this relationship between DNA/Lipid ratios and particle size has been observed, where higher lipid content leads to more compact particles [7,40]. LNP-Luc (40) had an average size of approximately 136 nm, while LNP-Luc (10) was larger at about 188 nm. We also demonstrated that adding PEI influenced size, although the extent depended on the method used. The two-step method, which involves preforming PEI/DNA complexes, resulted in greater variation in particle size. For example, LNP-Luc/PEI 1:1 (10) had a size range of 165–194 nm. In contrast, particles produced using the one-step method, where PEI was added directly to the organic phase, exhibited more consistent sizes.

Looking at the PDI values (Table 1), lower values, e.g., the 0.033 average seen in LNP-Luc (40), suggested a highly uniform particle population. In contrast, LNP-Luc (10), with a higher PDI value (0.213), showed greater variability in size. Our results also showed that the presence of PEI generally increased the PDI values, especially when using the two-step preparation method (e.g., 0.185–0.447). The PEI-free formulation, LNP-Luc (40), showed a relatively high zeta potential (~7 mV), likely due to its high lipid-to-DNA ratio. Increasing the DNA/PEI ratio led to higher zeta potentials, reaching 7.46 mV with LNP-Luc/PEI 1:3 (40). However, LNP-Luc/PEI (40, Org) and pre-complexed LNP-Luc/PEI (40) 1:1 and 1:2 exhibited lower zeta potentials than LNP-Luc (40), despite the addition of the positively charged PEI. This suggests that PEI is primarily incorporated inside the nanoparticle core rather than remaining exposed to the surface, reducing its contribution to surface charge. Additionally, charge screening by PEGylated lipids may further dampen zeta potential measurements. This trend is consistent with previous findings, for example, those by Navarro et al. [41], where PEI-containing phospholipid micelle-like nanoparticles exhibited a significant reduction in zeta potential from +31 mV to ~0 mV, attributed to PEI encapsulation and PEGylation shielding effects. The study by Song et al. [42] found lipid coatings on PEI/DNA complexes lowered apparent zeta potential through modifications to surface charge.

Cryo-TEM images revealed that LNP-Luc (40) exhibited a typical spherical structure, as seen in Figure 1 (bottom, panel A), while PEI-containing formulations displayed structural variations. LNP-Luc/PEI (40, Org) exhibited electron-dense cores (Figure 1, bottom, panel B), and some particles displayed multilamellar, onion-like structures (Figure 1, bottom, panel C), suggesting that PEI–lipid interactions influenced nanoparticle self-assembly. Similarly, LNP-Luc/PEI 1:3 (40) exhibited a pronounced onion-like multilamellar morphology (Figure 1, bottom, panel D), likely reflecting PEI-DNA complex formation affecting lipid layer organization. Such multilamellar structures are commonly observed in lipid-polymer hybrid nanoparticles and are indicative of complex internal organization rather than aggregation-driven instability [43,44,45].

### 3.3. Cellular Uptake of Luciferase DNA-LNPs

**Figure 2:**
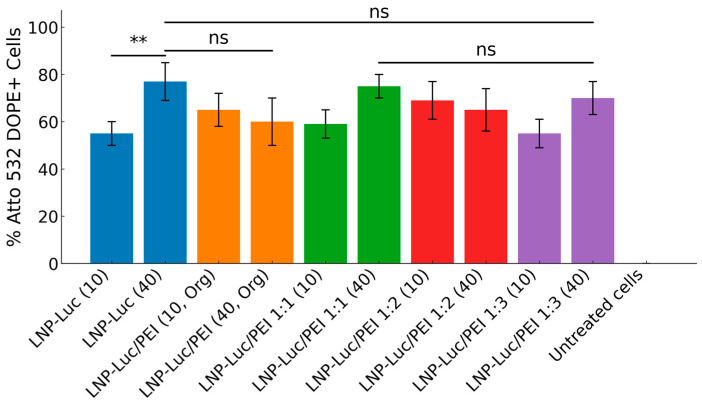
Cellular uptake of ATTO 532 DOPE-tagged formulations in HEK 293 cells after 4 h of incubation with 0.2 µg DNA per formulation, assessed by flow cytometry. Data show the mean ± standard deviation (SD) of three independent measurements. Statistical significance was determined using unpaired two-tailed *t*-tests for comparisons between two groups and one-way ANOVA for comparisons among three or more groups. Results are indicated as follows: ns (*p* > 0.05), ** (*p* < 0.01). Asterisks and “ns” annotations on the graph denote the significance of pairwise comparisons.

**Figure 3:**
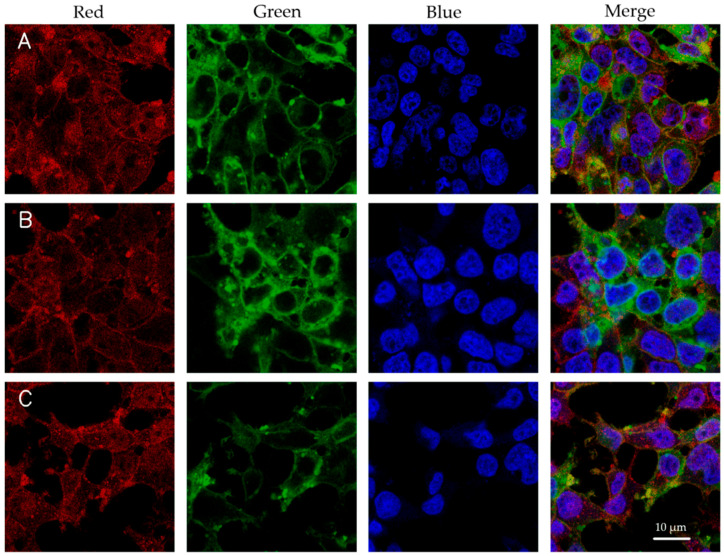
Confocal microscopy images of HEK 293 cells incubated for 4 h with ATTO 532 DOPE-tagged formulations (containing 0.5 µg DNA per treatment). DAPI was used to stain the nucleus (blue), and Alexa Fluor 633-WGA dye was used to label the cell membrane (red). (**A**) LNP-Luc (40), (**B**) LNP-Luc/PEI 1:2 (40), and (**C**) LNP-Luc/PEI (10, Org). The green fluorescence signal corresponds to ATTO 532 DOPE-labelled nanoparticles, indicating cellular uptake. Scale bar: 10 µm.

The cellular uptake data were evaluated through flow cytometry with ATTO 532 DOPE-tagged formulations, with three independent measurements. Cells untreated (no formulation) and cells incubated with ATTO-532 alone, exhibited negligible uptake (Figure 2). The cellular uptake data for the tested formulations showed variable uptake (42.9–85%). Among the tested formulations, higher DNA/Lipid ratios (1:40) generally improved cellular uptake compared to lower ratios (1:10). For example, LNP-Luc (40) exhibited a mean uptake of ~77%, substantially higher than the ~50% observed for LNP-Luc (10). At the lower lipid ratio (1:10), incorporating PEI into the formulations enhanced uptake. LNP-Luc/PEI 1:3 (40) achieved a mean uptake of ~76%, compared to formulations with lower PEI ratios, such as LNP-Luc/PEI 1:1 (40), which achieved ~69%. We found that both lipid and PEI ratios play a role in cellular uptake. To add further support, confocal microscopy was used to visualize the uptake of the same tagged formulations. Figure 3 presents confocal images of HEK 293 cells after 4 h of incubation, confirming the successful internalization of the nanoparticles and supporting the quantitative flow cytometry results.

### 3.4. Cell Viability and Luciferase Expression In Vitro

**Figure 4:**
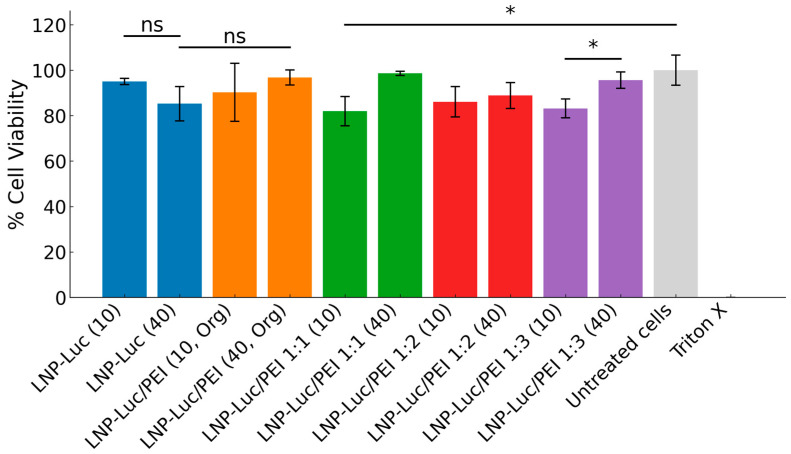
Cell viability of HEK 293 cells treated with various formulations (MTT assay). Untreated cells served as the control (100% viability). Triton X was used as a positive control to confirm complete cell death (0% viability). Statistical significance was determined using unpaired two-tailed *t*-tests for comparisons between two groups and one-way ANOVA for comparisons among three or more groups. Results are indicated as follows: ns (*p* > 0.05) and * (*p* < 0.05). Asterisks on the graph denote significant differences between groups.

**Figure 5:**
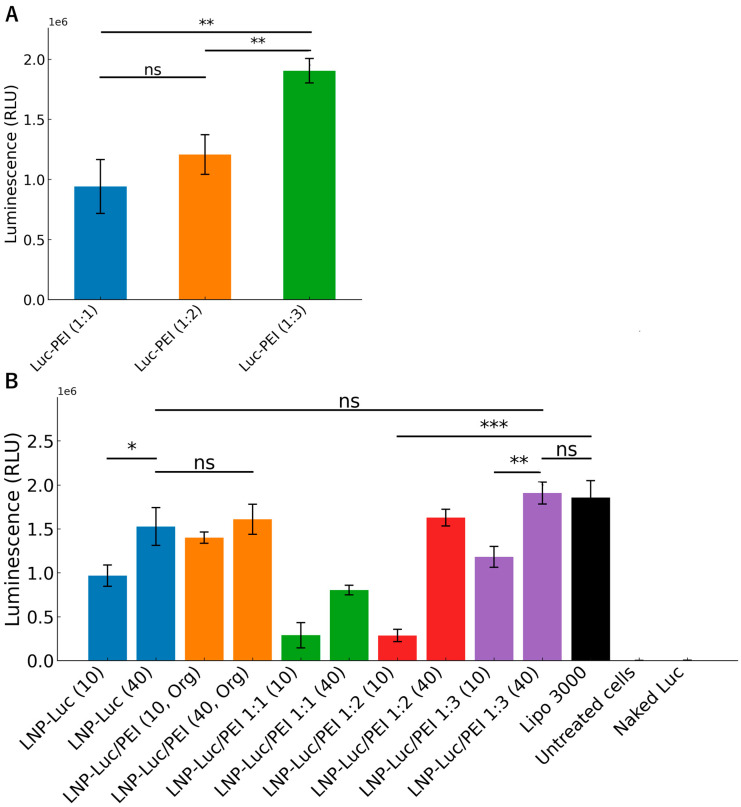
(**A**) Luciferase expression of precomplexed Luc-PEI formulations with different DNA/PEI ratios (1:1, 1:2, 1:3), measured as luminescence intensity (RLU) in HEK 293 cells. (**B**) Luminescence intensity (RLU) of various lipid-based formulations in HEK 293 cells after 48 h of incubation with a DNA dose of 0.2 µg. Error bars represent the standard deviation from three independent replicates. Statistical significance was determined using unpaired two-tailed *t*-tests for comparisons between two groups and one-way ANOVA for comparisons among three or more groups. Results are indicated as follows: ns (*p* > 0.05), * (*p* < 0.05), ** (*p* < 0.01), and *** (*p* < 0.001). Asterisks on the graph denote significant differences between groups.

We conducted cell viability studies that showed that all formulations were well-tolerated by the cells. The formulations with higher DNA/lipid ratios (1:40) generally maintained better cell viability compared to their lower ratio counterparts (1:10) (Figure 4). LNP/PEI 1:1 (40) showed the highest cell viability at >100%, comparable to untreated cells, while LNP/PEI 1:1 (10) had the lowest viability at 87.0%. The luminescence data demonstrate the efficiency of different formulations in delivering the luciferase plasmid DNA to HEK 293 cells after 48 h of incubation. Luminescence intensity (measured in RLU) reflects the level of gene expression, providing a quantitative measure of transfection efficiency. We found that precomplexed PEI-Luc complexes exhibited a dose-dependent increase in luciferase expression with increasing DNA/PEI ratios (Figure 5A). Luc-PEI formulations with DNA/PEI ratios of 1:1, 1:2, and 1:3 yielded mean luminescence values of 9.4 × 10^5^, 1.2 × 10^6^, and 1.9 × 10^6^ RLU, respectively.

We found that increasing the lipid content in the formulations improved their performance in luminescence assays. For example, LNP-Luc (40) showed higher luminescence than LNP-Luc (10), which had less lipid content. This suggests that higher lipid ratios may enhance DNA encapsulation and delivery efficiency. The addition of PEI further improved performance—both LNP-Luc/PEI (10, Org) and LNP-Luc/PEI (40, Org) exhibited higher luminescence than their PEI-free counterparts (Figure 5B). The ratio of DNA to PEI also contributed significantly. Formulations with higher PEI content (DNA/PEI ratios of 1:2 and 1:3) achieved much higher luminescence levels than those with a 1:1 ratio.

### 3.5. Development of GFP-Based Formulations

After successfully developing luciferase-based formulations, we extended our approach to GFP-based systems. Table 2 shows how adding PEI affected the physicochemical properties of the LNP-GFP formulations. Without PEI, the LNP-GFP (40) formulation had the smallest particle size (69.17 ± 1.30 nm) and a near-neutral zeta potential (0.12 ± 1.61 mV), reflecting minimal surface charge. Adding PEI at a 1:1 ratio (LNP-GFP/PEI 1:1) increased the particle size to 119.53 ± 7.75 nm and made the zeta potential more positive (3.43 ± 2.00 mV), consistent with PEI’s cationic nature. Increasing the PEI ratio to 1:3 (LNP-GFP/PEI 1:3) resulted in larger particles (183.03 ± 17.42 nm) and a higher zeta potential (12.67 ± 0.57 mV). Importantly, all formulations exhibited relatively low PDI values (≤0.23), which may suggest a degree of heterogeneity in particle populations.

**Figure 6:**
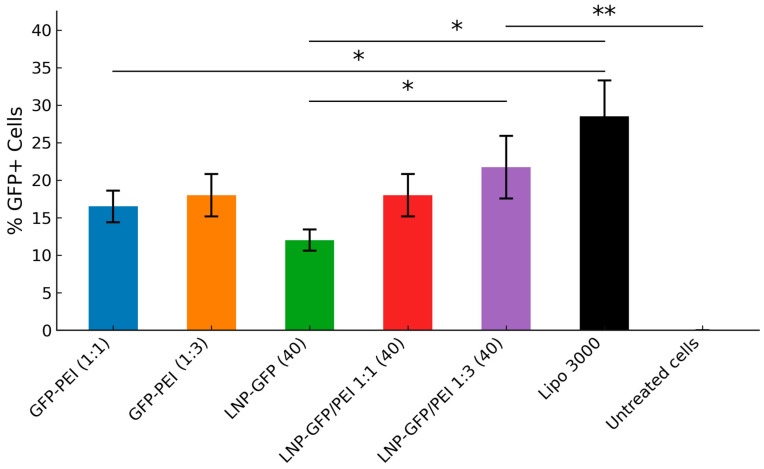
Percentage of GFP-Positive Cells in HEK 293 Transfection Study. Bar graph showing the percentage of GFP+ cells measured by flow cytometry after transfection with different DNA-lipid formulations. We transfected HEK 293 cells with 0.2 µg DNA and incubated them for 48 h. Error bars represent the standard deviation from independent replicates. Statistical significance was determined using unpaired two-tailed *t*-tests for comparisons between two groups and one-way ANOVA for comparisons among three or more groups. Results are indicated as: * (*p* < 0.05), and ** (*p* < 0.01). Asterisks denote significant differences between groups.

**Figure 7:**
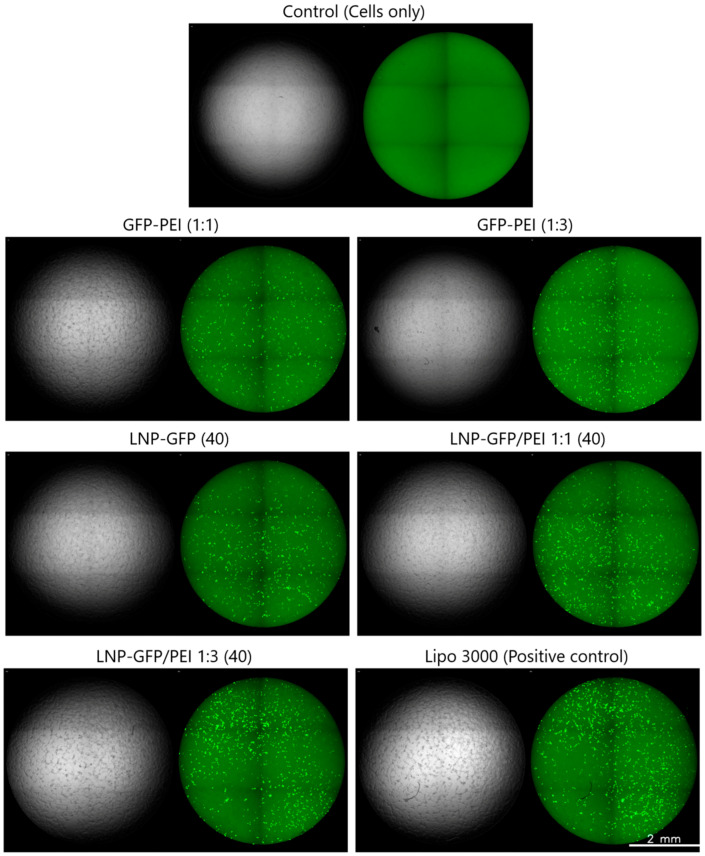
GFP expression in HEK 293 cells following transfection. Fluorescence microscopy images of HEK 293 cells 48 h post-transfection, with each image showing a bright field (**left**) and a green fluorescence (**right**) view side by side. Cells were transfected with lipofectamine 3000 or experimental GFP-based formulations at a dose of 0.2 µg DNA. The control represents untransfected cells (no DNA or transfection agents added).

We observed varying transfection efficiencies across our GFP formulations (Figure 6). Increasing the PEI ratio in the precomplexed (GFP-PEI) formulations had little effect, (1:1, 17% and (1:3, 19% GFP-positive cells). In the LNP group, there was a PEI-ratio dependent increase in transfection efficiency. Moreover, the LNP-PEI (1:3) formulation had a higher efficiency than GFP-PEI (1:3), indicative of LNP having an additive effect at high PEI ratios. The positive control, Lipo 3000, achieved ~30% GFP-positive cells. We used fluorescence microscopy to visualize GFP expression in the treated HEK 293 cells (Figure 7). Using the ZEISS Celldiscoverer 7 system for live-cell imaging, we observed successful transfection with all our formulations. The images clearly showed the differences between untreated control cells and those treated with either Lipo 3000 or our test formulations. More importantly, cells treated with LNP-GFP/PEI 1:1 (40) and LNP-GFP/PEI 1:3 (40) showed stronger fluorescence signals (fluorescence intensity: 8.6 ± 0.4 × 10^6^ A.U. and 9.9 ± 0.4 × 10^6^ A.U., respectively) than those treated with LNP-GFP (40) (5.2 ± 0.3 × 10^6^ A.U.), supporting our earlier flow cytometry results.

## 4. Conclusions

Our study developed PEI-enhanced LNPs through the systematic optimization of formulation parameters and preparation methods to achieve efficient DNA delivery. Adding PEI improved cellular uptake and transgene expression in luciferase formulations. The highest transfection efficiency (~1.9 × 10^6^ RLU) was achieved with a DNA/PEI ratio of 1:3. Formulations with higher DNA/lipid ratios of 1:40 generated smaller particles (136 nm vs. 188 nm) and achieved better cellular uptake (77% vs. 50%) compared to lower ratios of 1:10. We also found that particle properties depended on the preparation method used. The one-step technique for luciferase formulations without PEI generated uniform particles, as demonstrated by LNP-Luc (40), with a PDI of ~0.034 and a particle size of 136.27 ± 1.69 nm.

Adding PEI generally maintained good size consistency (e.g., LNP-Luc/PEI (10, Org), PDI 0.083 ± 0.036), though two-step formulations with higher PEI content showed increased polydispersity (PDI > 0.3), indicating greater particle heterogeneity. PEI incorporation also increased zeta potential to positive values, reaching up to 7.46 mV in the 1:3 ratio formulation. All luciferase formulations showed both efficacy and safety. They kept cell viability above 87%. The 1:40 formulations were particularly biocompatible. For example, LNP-Luc/PEI 1:1 (40) showed over 100% viability. Validation with a complementary reporter system such as GFP further confirmed enhanced transgene expression. LNP-GFP/PEI (1:3) achieved ~23.75% GFP-positive cells, approximately twofold higher than that achieved with PEI-free formulations (~12.6%). Further research should examine diverse DNA/PEI and DNA/lipid ratios to improve distinct formulation attributes, e.g., stability, particle size, and transfection efficiency. Additional mechanistic studies on intracellular trafficking and endosomal escape would give a better understanding of the enhanced transfection observed. In vivo evaluation would also help examine biodistribution and safety, supporting the clinical translation of PEI-enhanced LNPs.

## Figures and Tables

**Table 1:** LNP formulations, preparation methods, and physicochemical properties. All formulations contain plasmid DNA encoding luciferase and the same lipid composition (mole percent, %): DOTAP: 50%; DSPC: 10%; Cholesterol: 38.5%; DMG-PEG 2000: 1.5%. “Org” indicates PEI addition to the organic phase in the one-step method. Formulations were prepared using the NanoAssemblr microfluidic system. Hydrodynamic diameter (HD, nm), polydispersity index (PDI), and zeta potential (mV) are shown as mean ± SD.

Formulation	DNA/Lipid (*w*/*w*)	DNA/PEI (*w*/*w*)	Size (nm, Mean ± SD)	PDI (Mean ± SD)	Zeta Potential (mV, Mean ± SD)	Preparation Method
LNP-Luc (10)	1:10	-	188.8 ± 3.88	0.213 ± 0.018	0.068 ± 0.091	One-step (NanoAssemblr direct)
LNP-Luc (40)	1:40	-	136.27 ± 1.69	0.034 ± 0.009	6.003 ± 0.357	One-step (NanoAssemblr direct)
LNP-Luc/PEI (10, Org)	1:10	1:1	200.4 ± 3.78	0.083 ± 0.036	0.155 ± 0.067	One-step (PEI in organic phase, NanoAssemblr)
LNP-Luc/PEI (40, Org)	1:40	1:1	109.9 ± 2.02	0.144 ± 0.021	0.225 ± 0.058	One-step (PEI in organic phase, NanoAssemblr)
LNP-Luc/PEI 1:1 (10)	1:10	1:1	177.4 ± 11.6	0.325 ± 0.029	0.313 ± 0.162	Two-step (PEI/DNA complex, NanoAssemblr)
LNP-Luc/PEI 1:1 (40)	1:40	1:1	111.07 ± 4.64	0.195 ± 0.008	0.622 ± 0.244	Two-step (PEI/DNA complex, NanoAssemblr)
LNP-Luc/PEI 1:2 (10)	1:10	1:2	250.2 ± 22.6	0.300 ± 0.018	0.016 ± 0.009	Two-step (PEI/DNA complex, NanoAssemblr)
LNP-Luc/PEI 1:2 (40)	1:40	1:2	201.4 ± 14.9	0.289 ± 0.023	0.184 ± 0.023	Two-step (PEI/DNA complex, NanoAssemblr)
LNP-Luc/PEI 1:3 (10)	1:10	1:3	182.1 ± 18.4	0.379 ± 0.049	0.336 ± 0.086	Two-step (PEI/DNA complex, NanoAssemblr)
LNP-Luc/PEI 1:3 (40)	1:40	1:3	154.6 ± 12.6	0.254 ± 0.013	7.46 ± 0.833	Two-step (PEI/DNA complex, NanoAssemblr)

**Table 2:** Physicochemical properties of GFP-based formulations, including preparation methods, DNA ratios, particle size (nm), polydispersity index (PDI), and zeta potential (mV). Values represent the mean ± SD.

Formulation	DNA/Lipid (*w*/*w*)	DNA/PEI (*w*/*w*)	Preparation Method	Size (nm, Mean ± SD)	PDI (Mean ± SD)	Zeta Potential (mV, Mean ± SD)
LNP-GFP (40)	1:40	-	One-step (NanoAssemblr direct)	69.17 ± 1.30	0.23 ± 0.04	0.12 ± 1.61
LNP-GFP/PEI 1:1 (40)	1:40	1:1	Two-step (PEI/DNA complex, NanoAssemblr)	119.53 ± 7.75	0.20 ± 0.01	3.43 ± 2.00
LNP-GFP/PEI 1:3 (40)	1:40	1:3	Two-step (PEI/DNA complex, NanoAssemblr)	183.03 ± 17.42	0.23 ± 0.01	12.67 ± 0.57

## Data Availability

The data supporting the reported results are available upon request from the corresponding author.

## Supplementary Materials

The following supporting information can be downloaded at: https://www.mdpi.com/article/10.3390/pharmaceutics17040454/s1, Figure S1: Original, uncropped Cryo-TEM images corresponding to Figure 1 (PDF).

## Abbreviations

The following abbreviations are used in this manuscript:

ANOVA	Analysis of variance
DAPI	4′,6-diamidino-2-phenylindole
DMG-PEG2000	1,2-dimyristoyl-rac-glycero-3-methoxypolyethylene glycol-2000
DMSO	Dimethyl sulfoxide
DNA	Deoxyribonucleic acid
DOPE	Dioleoylphosphatidylethanolamine
DOTAP	1,2-dioleoyl-3-trimethylammonium-propane
DSPC	1,2-distearoyl-sn-glycero-3-phosphocholine
EE	Encapsulation efficiency
GFP	Green fluorescent protein
HD	Hydrodynamic diameter
LB	Lysogeny broth
LNPs	Lipid nanoparticles
MTT	3-(4,5-Dimethylthiazol-2-yl)-2,5-Diphenyltetrazolium Bromide
PAMAM	Polyamidoamine
PBS	Phosphate-buffered saline
PDI	Polydispersity index
PEI	Polyethyleneimine
PFA	Paraformaldehyde
RLU	Relative light units
SEM	Standard error of the mean
siRNA	Small interfering RNA
TE	Tris-EDTA
