# Supplementary material for: Microfluidic Optimization of PEI-Lipid Hybrid Nanoparticles for Efficient DNA Delivery and Transgene Expression
Source: Pharmaceutics. 2025 Apr 1;17(4):454. doi: 10.3390/pharmaceutics17040454 (PMC12030462; doi:10.3390/pharmaceutics17040454)
Supplement: Supplementary file 1 [file pharmaceutics-17-00454-s001.zip › pharmaceutics-3522828-supplementary.pdf]

Original Microscopy Images – Supporting Figures

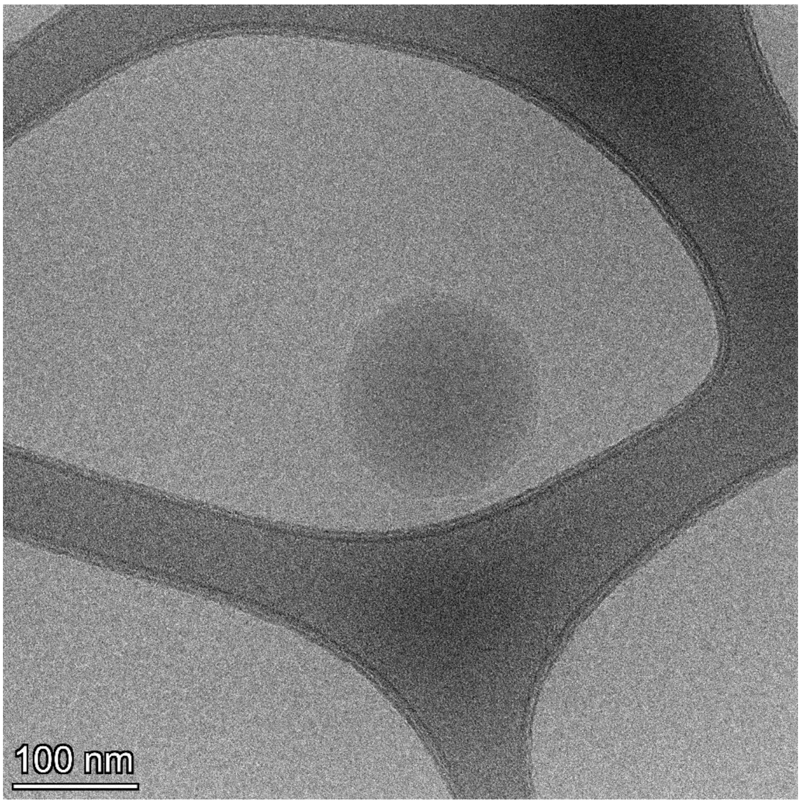

Figure S1A

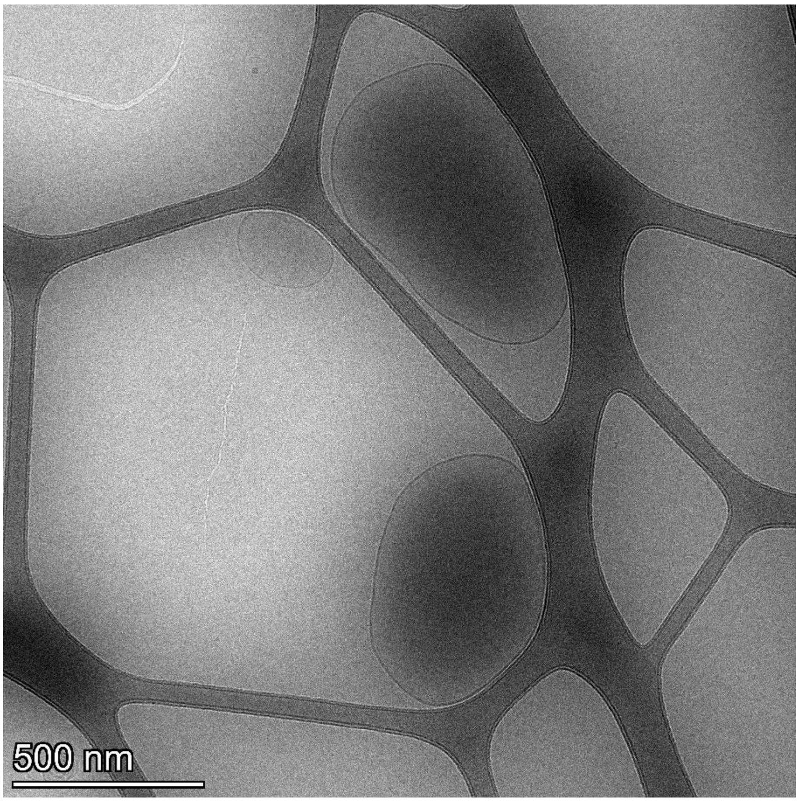

Figure S1B

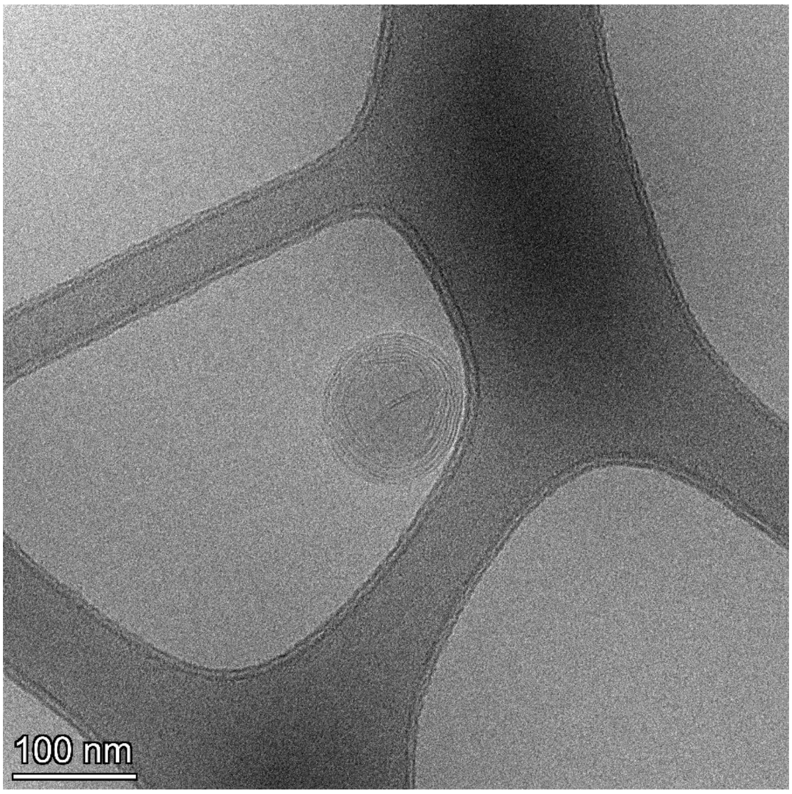

Figure S1C

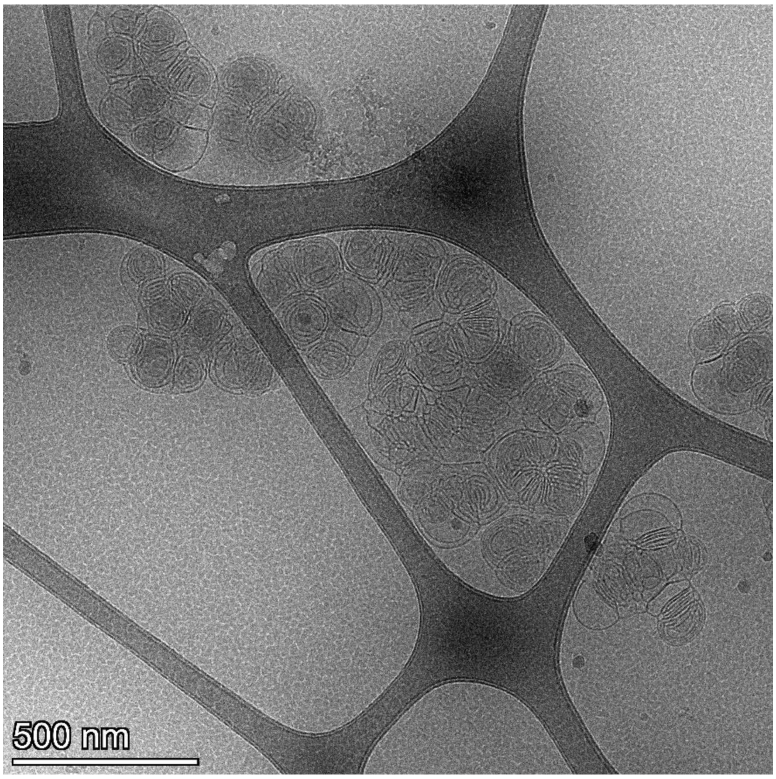

Figure S1D

Figure S1. Original, unprocessed Cryo-TEM images of LNP formulations. These images correspond to Figure 1 in the main manuscript.

Note:

The **cell uptake** and **GFP expression** images presented in the main manuscript are already original and unprocessed. Therefore, they are not included in this supplementary file, as per journal guidelines.
